# Good Practices in Home Kitchens: Construction and Validation of an Instrument for Household Food-Borne Disease Assessment and Prevention

**DOI:** 10.3390/ijerph16061005

**Published:** 2019-03-20

**Authors:** Adenilma da Silva Farias, Rita de Cassia Coelho de Almeida Akutsu, Raquel Braz Assunção Botelho, Renata Puppin Zandonadi

**Affiliations:** 1 Federal Institute of Piauí, Campus Pedro II, Piauí 64255-000, Brazil; adenilma.farias@ifpi.edu.br; 2 Department of Nutrition, Faculty of Health Sciences, University of Brasilia, Brasilia 70910-900, Brazil; rita.akutsu@gmail.com (R.d.C.C.d.A.A.); raquelbabotelho@gmail.com (R.B.A.B.)

**Keywords:** good practices, home kitchens, foodborne disease, prevention, instrument

## Abstract

This study aimed to develop and validate an instrument to evaluate Brazilian home kitchens’ good practices. We elaborated on the preliminary version of the check-list based on the Brazilian resolution for food safety Collegiate Board Resolution 216 (RDC 216), Collegiate Board Resolution 275 (RDC 275), the standard 22000 from the International Organization for Standardization (ISO 22000) and Codex Alimentarius. Seven experts with experience in the area participated in the check-list validation and semantic evaluation. The criteria used for the approval of the items, as to their importance for the prevention of food contamination and clarity of the wording, was the achievement of a minimum of five out of seven of agreement among the experts (*W*-values ≥ 0.7). Moreover, items should have a mean ≥3 for the evaluation of importance (content validation) and clarity (semantic evaluation) to be maintained in the instrument. After the expert phase, we conducted another semantic evaluation of the check-list with a focus group composed of 13 undergraduate students, one moderator, and one observer of the process, to evaluate each item regarding its clarity, considering their level of understanding of the item. The final version of the check-list was composed of 77 items, divided into four blocks. The check-list developed was validated with respect to content with a W-value of 0.86 and approved in the semantic evaluation.

## 1. Introduction

Foodborne diseases (FBD) represent one of the most common and important public health problems in the world, being one of the major causes of personal distress, preventable deaths, and avoidable economic burden [1]. Despite the considerable global burden of FBD, the full extent of unsafe food and its damage to the public has been unknown [2,3]. Every year, at least two billion people worldwide suffer from FBD, which makes these diseases among the greatest public health problems in the contemporary world. Data from Canada’s health agency show that in 2016, 1.6 million people became ill, 4000 were hospitalized, and 105 died from FBD in that country [4]. According to the World Health Organization (WHO) [5], 23 million people in Europe become ill, and 5000 die from FBD every year. The U.S. reported about 39 million cases of FBD per year, with about 70,000 hospitalizations and about 1600 deaths [6,7]. Data from Brazil showed about 13,000 individuals with FBD, 1700 hospitalized per year [2].

According to the U.S. Centers for Disease Control and Prevention, food consumed at home causes about 20% of FBD [8]. According to data from the epidemiological analysis of FBD outbreaks, from 2000 to 2011 in Brazil, 51.8% of the outbreaks arose in households, more than in restaurants [9,10]. The occurrence of FBD outbreaks in residences shows the population lacks sanitary education and knowledge about adequate preparation and storage of food [11]. The percentage of cases due to unsafe food preparation practices in households can be linked to the food handler, by using poor hygiene, improper food storage conditions, and inadequate meal preparation practices [10].

We could avoid many FBD cases if people adopted preventive actions throughout the home food production chain. Therefore, proper food handling in households represents an important step in reducing the incidence of FBD. In this sense, the population should know and adopt the good practices to guarantee food quality and safety, avoiding the FBD occurrence [12,13].

Despite the concerns about FBD at home, research about home food safety practices is still scarce [10]. Government regulates food safety practices in commercial establishments by the laws which provide the main means of intervention for municipal health surveillance. However, there is little health surveillance of food safety at homes in Brazil. This lack of action affects a large number of homes and undermines health surveillance, which includes the inspection of locations that may present a risk to public health [10]. Moreover, considering the concerns about the increase in the cases of FBD arising in households, there is a need to develop an instrument to evaluate good food preparation practices. It is an important strategy for reducing FBD cases to improve food handling practices in household environments. Therefore, this study aimed to develop and validate an instrument to evaluate home kitchens’ good practices.

## 2. Materials and Methods

### 2.1. Items Construction

The instrument (check-list) was elaborated based on extensive literature review and experience of the researchers on the matter. The following documents were used to design the preliminary version of the check-list: Brazilian legislation as RDC No. 216/04 [14], RDC No. 275/02 of the National Health Surveillance Agency-ANVISA [15], and international standards ISO 22000 [16] and Codex Alimentarius [17]. After reviewing the literature, we found the following critical indicators that should be considered in the analysis of hygiene conditions in home kitchens:

(a) Building and facilities;
(b) Kitchen equipment, furniture, and utensils;
(c) Manipulators;
(d) Raw materials and ingredients;

Each of the indicators had subitems, and we evaluated each subitem according to the following criteria—“attend,” “do not attend,” and “do not apply”—as well as studies from Ceniccola, Araujo, and Akutsu [18] and Araujo et al. [19]. In the end, we obtained 82 subitems that were part of the four critical indicators.

### 2.2. Content Validation

We used the Delphi method, with some adjustments for the content validation. This method is based on obtaining the opinions of experts to achieve a consensus on a specific subject. The Delphi method is currently employed in several areas in situations where new ideas are being created. It is a method in which, through collegial communication ordered by individual responses, often conducted by questionnaires, we seek the consensus of a group [20].

We used the Survey Monkey^@^ platform (Surveymonkey, San Mateo, CA, USA) to create a questionnaire for the application of the check-list content validation. On the first page of the questionnaire, there was an orientation letter specifying the evaluation criteria for the check-list items. We asked experts specialized in food hygiene and food safety (Ph.D. and/or post-graduate professors with extensive experience in the research field) to evaluate each item considering its pertinence using a Likert scale, as follows: (0) “I totally disagree with the item”; (1) “I partially disagree with the item”; (2) “I neither agree nor disagree with the item”; (3) “I partially agree with the item”; and (4) “I fully agree with the item.”

We also used the Survey Monkey^@^ platform to provide feedback to the experts regarding the evaluations performed by other experts and the results of the analysis. We conducted two evaluation stages in the content validation process. For the items which did not receive approval in the first stage, we presented to each one of them the collection resulting from the experts’ opinions. After being informed about the other experts’ opinions, the experts were asked to review their analysis and decide whether they would confirm previous answers. We conducted this procedure to obtain consensus among the experts. Seven experts participated in this phase. We confirmed each item for the final instrument when it received 70% of approval by the experts.

### 2.3. Semantic Evaluation

We performed a semantic evaluation of the check-list simultaneously with the content validation with the same experts, using the same questionnaire in the Survey Monkey^@^ platform. For that purpose, we used the Likert scale, as follows: (0) “I did not understand it at all”; (1) “I understood it a little”; (2) “I somewhat understood it”; (3) “I understood almost everything, but I had some questions”; (4) “I understood almost everything”; (5) “I understood it perfectly and had no questions.” According to Conti et al. [21], answers from 0 to 3 indicate insufficient understanding and a new version of the item is required [21].

After the expert phase, we conducted another semantic evaluation of the check-list. We asked 13 undergraduate students to evaluate each item regarding its clarity, considering their level of understanding of the item. We conducted a focus group with these students, using one moderator and one observer of the process. For that purpose, we used the same Likert scale of the experts.

In cases of poor understanding of the item or unsuitable language, we asked students to suggest changes. We used these commentaries to create new versions of the items for a second evaluation. We needed two evaluation stages for the semantic process with the students. These stages were necessary to have a comprehensive instrument that could be applied by trained people and not only experts in the field.

### 2.4. Data Analysis

We used the Software Excel 97–2003 (Microsoft, Redmond, DC, USA) and SPSS for Windows (version 21, SPSS Inc., Chicago, IL, USA) for data analysis. We calculated the average score for the evaluation of the importance and clarity of each item considering the answers provided by the seven specialists. We assessed the degree of agreement between the experts to assess the importance and clarity of the items using the Kendall coefficient (*W*) of the agreement, which ranges from 0 to 1. High values of *W* (*W* ≥ 0.66) indicate that the specialists applied the same evaluation standards in comparison with the low *W*-values, which suggest disagreement between them [22,23]. The criteria established for the approval of the item was a minimal of 70% of agreement between the experts (*W*-values ≥ 0.7).

Moreover, items should have a mean ≥3 for the evaluation of importance (content validation) and clarity (semantic evaluation) to be maintained in the instrument. We excluded from the instrument items not considered important for the prevention of food contamination in households. We rewrote items considered unclear in a different manner and subject to further evaluation by the experts. Suggestions made by the experts were considered and incorporated into the final version of the instrument.

Brasilia University Research Ethics Committee approved the project before the tests (CAAE: 23955313.3.0000.0030).

## 3. Results

**Figure 1:**
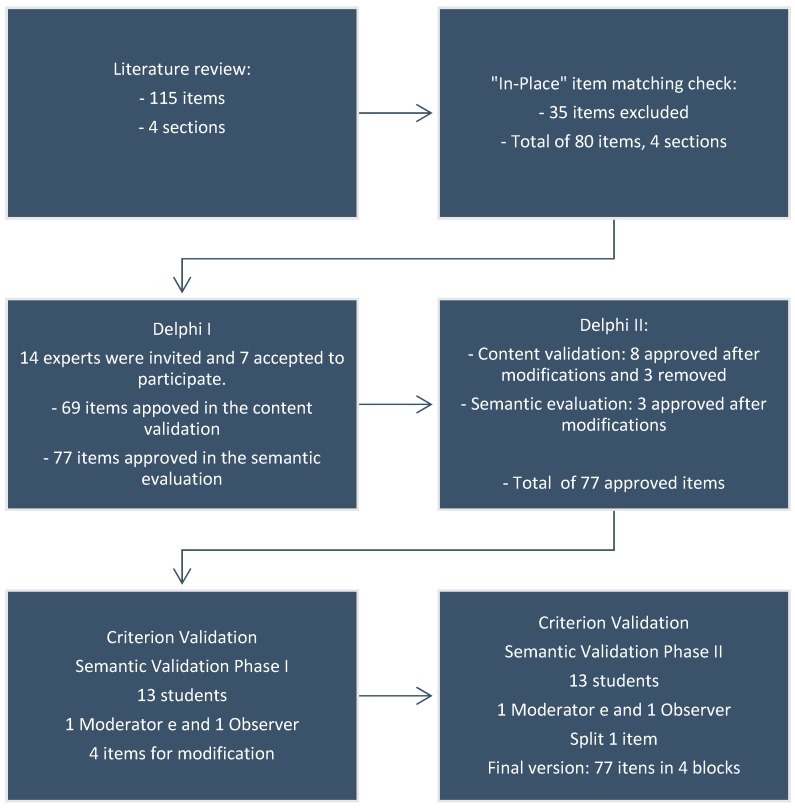
Stages of the instrument content validation and semantic evaluation.

After reviewing the literature, we constructed a checklist-type instrument with 115 items. We conducted the “in-place” item matching check (in 10 households in five different regions of the Federal District—Brazil) and excluded 35 items based on the pertinence after this phase. According to Pasquali [24], in a pilot study, ten households are enough for an in-place item match check. Moreover, this pilot test was useful to define the checklist application duration. Therefore, we created a new version of the instrument composed of 80 items, divided into four blocks, with the reorganization of the items within the blocks. This new instrument version was then subjected to an objective evaluation by specialists. We carried out the objective evaluation with the accomplishment of content validation and semantic evaluation using the Delphi Technique in two phases, and the validation of the criterion, using the semantic validation in two stages. Figure 1 shows a summary of the checklist validation steps.

### 3.1. Content Validation and Semantic Evaluation by Experts

#### 3.1.1. Phase Delphi I

We used the Delphi technique for content validation. We invited 14 judges specialized in food hygiene and safety; however, only seven participated up to the last phase.

We divided the instrument into four blocks. The first refers to the building and facilities that affect the production of food in the residences; the second refers to equipment, furniture, and kitchen utensils; the third refers to handlers; and the fourth block refers to the ingredients used in food preparation.

In the first round, about the first block of the instrument, the experts suggested the revision of the item that dealt with the external area of the kitchens (item 1.1). Thus, the item that was written “Access roads with a hard or paved surface, suitable for traffic on wheels, adequate and clean flow; adequate sewage system” changed to “Access roads with adequate sewage system”. Still in the first block, the experts suggested adding a question about the type of material of the kitchen floor (item 1.3), they also suggested adding a question for the material of the roof of the residences (item 1.4).

The experts suggested adding the expression “constructed with material that facilitates its cleaning” to the items related to walls, doors, windows, and other openings (item 1.5 of the questionnaire).

In item 1.8, the term “toilet facilities” was replaced by “toilet”. In section 1.11, we rewrote the text “Adoption of preventive and corrective measures with the objective of preventing attraction, shelter, access, and/or proliferation of vectors and urban pests” to “Implementation of disinfecting every six months”.

In item 2.1, we changed “Food storage equipment (refrigerators, freezers, and others), as well as those designed to the thermal processing (stove), in proper operation” to “Food storage equipment (refrigerators, freezers, and others), in proper functioning”. In Section 2.3, we changed “Non-contaminating material, resistant to corrosion, of size and shape that allow easy hygiene: in good condition” to “In size and shape that allows easy hygiene: in good condition”. We added to the item 2.3, “No utensils made of wood or other easily contaminated material” as requested by the judges. The judges also requested the inclusion of an item that talked about the storage of cleaning materials (item 2.4).

In item 4.2, the experts recommended adding the question “Food served immediately after preparation or, if prepared in advance, reheated before being served”. It is important to emphasize that Costa et al. [25] showed that in Brazil lunch leftovers usually are left on the kitchen counter until dinner time, increasing the risk of FBD. Therefore, the experts judged enough the sentence proposed without the timeline information.

In the first phase of the content validation and comprehension (Delphi I) process, 69 items (86.25%) were approved by the experts regarding content validation, and 77 items (96.25%) were considered comprehensible and; therefore, approved without the need of text adjustment.

#### 3.1.2. Phase Delphi II

In the second round of the Delphi Phase, experts considered items 1.1, 1.3, 1.4, 1.5, 1.8, 1.11, 1.12, 2.1, 2.3, 2.4 and 4.2 approved after the review. With all the experts agreeing on the items, we ended the objective content validation step.

In this phase, we reformulated the items that did not obtain a minimum grade for approval (≥3) in the first stage and presented again for the same experts. At the end, we obtained an instrument with 77 items, divided into 4 blocks. Table 1 shows the average values of the notes and W-values obtained for each item, by block and by the set of blocks, in content validation and semantic validation after the completion of all steps.

### 3.2. Criteria Validation

For validation of the criterion, we used semantic analysis with a focus group, in a 2 h meeting conducted by the researcher. We had a moderator (researcher), an observer (Ph.D. student in Human Nutrition), and 13 undergraduate students in Nutrition at the University of Brasília (Brazil) in the group.

The group consisted of thirteen students (male: *n* = 6; 46%). The inclusion criterion was to accept participation in the research and to have studied the courses of hygiene and food legislation. The members of the group were aged between 20 and 28 years (mean: 22.9 ± 2.99) and were in the fifth to the eighth term of the Nutrition undergraduate course.

In the first phase, despite being considered fully understandable, the group suggested that in item 1.2, belonging to the Building and Facilities block, only the expression “Free of the presence of domestic animals” should remain. Additionally, they suggested to specify items 1.5, 1.6, and 1.7 better, where “Constructed of material that facilitates its cleaning” was replaced by “Constructed of material that facilitates its cleaning (plane, without roughness and cracks)”.

In the second phase, they suggested dividing item 4.2 (dealing with food storage). Thus, what was previously described “The ingredients that were not fully utilized are stored in clean and closed containers and identified with shelf life after the opening.” was revised and rewrote as follows: “Ingredients that have not been fully utilized are stored in clean, closed containers.” and “Ingredients that have not been fully utilized are identified as shelf-life after opening”.

## 4. Discussion

It is very important to use rigorous methods in the process of development and validation of an instrument [18]. In our study, we used the Delphi technique, which allows the implementation of an expert’s panel to perform the content validation, facilitating the achievement of consensus on the experts’ opinions [26]. The Delphi technique was used to guide the stages of the experts’ evaluations, making them interact with the research group through structured rounds [18,26]. We performed the research using the Survey Monkey^@^ platform, which enables the provision of feedback to the experts, since the feedback is proposed in the Delphi technique to assure a more organized interaction with the experts [18]. After this stage, we used an analysis with a focus group for validation of the criterion, since the construction of a measuring instrument requires the design of the items that represent the construct of interest [27]. We reformulated the contents and format of the items according to the contributions of specialists and after the focus group (Figure 1). The use of the focus group technique allowed relevant changes in the items and promoted ideas of how the items should be displayed to the best comprehension [27,28].

In the first phase (experts’ evaluation), the appropriate selection of the experts is also a critical point to obtain solid results, and it is based on the experience and the knowledge of the participants in a certain area, besides the willingness to collaborate with the study. In our study, we invited postgraduate experts (Ph.D. or MSc individuals) that work with instruments and food safety or foodborne diseases. Despite the fact that there is no consensus in the literature in regards to the number of experts to perform the validation process, Pasquali [24] considers that a minimum of six experts is necessary to reach a consensus, although this number may vary according to the type of the instrument. In our study, a total of 14 experts were invited, and seven experts participated up to the final phase of experts content and comprehensive validation.

The rules defined in resolutions on the subject have proved to be effective, since studies have mentioned the reduction of outbreaks of foodborne diseases caused by food eaten out of home [10,29,30,31]. According to Draeger et al. [2], in Brazil, the analysis of the initial sites of outbreaks showed that the places with the highest occurrence were residences, with 2922 cases (38.3%) from 2007 to 2017. However, there is a lack of studies on the development of quality control instruments for the prevention of households cross-contamination and FBD. The occurrence of FBD outbreaks in residences shows a serious problem regarding the lack of sanitary education, and knowledge about adequate preparation and storage of food by the population in general [2,11,32]. Outbreaks of FBD in households tend to be less well-known because they involve a smaller number of people (usually family). This fact contributes to the lack of direction in educational campaigns and training for this public [2,3,33].

In this study, we elaborated and evaluated a check-list with the purpose of providing an appropriate tool to assist the household food production and to reduce the FBD outbreaks, since a large proportion of the people who prepare food at home are poorly informed about the measures required to prevent foodborne diseases [31]. Consumers tend to do not perceive themselves, or someone in their family, to be susceptible to foodborne illness, rank their risk of foodborne illness lower than that of others, and/or do not follow all recommended food safety practices, and, consequently, they do not take sufficient precautions [34,35,36,37]. In block three of our instrument, the evaluation refers to the “handler’s” hygiene habits and health condition. Hands are the most important vehicle for spreading pathogens in the kitchen [11,37]. Therefore, the washing hand procedure is critical to preventing cross-contamination. Many handlers wrongly wash their hands to clean them, ignoring the possibility of contamination. It is important to highlight that in item 3.1, the experts considered the item enough to understand. Additionally, there is no sink exclusive for washing-hands in a domestic kitchen in Brazil. Moreover, it is likely that hands are not washed frequently enough to prevent the transfer of pathogens to ready-to-eat food, food packaging, or equipment and contact surfaces used to prepare food. It is important to evaluate and also to show to the handlers the need to wash their hands after handling uncooked meats, fresh unwashed vegetables, after using the toilet, after touching dirty clothes, and playing with or touching animals/domestic animals.

Food preparation at home involves heavily contaminated areas in the kitchen (refrigerator handles, tap handles, sink drain areas, dishcloths, and sponges) because it is not common to wash or clean these areas frequently. Additionally, raw or unwashed foods are constantly touched during meal preparation. The modifications made on items from block one (Construction and Facilities) and two (Equipment, furniture, and kitchen utensils) were necessary since, unlike commercial enterprises, home kitchens are multipurpose areas, more than just food preparation places. Unfortunately, still in the present days, pets, papers, dirty laundry, and house plants are common at home kitchens or near them. In this sense, in item 1.2, the expression “Free of the presence of domestic animals” and “Kitchen free of obsolete objects or strangers to the environment” remained. Kitchen sinks are usually used for hand washing, dishwashing, food washing, kitchen cloth washing, pets pot washing, and support for a newly purchased package of food. Raw and unwashed vegetables, dripping raw meat, as well as cooked ready-to-eat foods are common in home refrigerators. The uses of home kitchens provide potential risk to introduce pathogens that can spread to foods, proliferate, and result in FBD [37]. Therefore, we carefully revised the final check-list with the intention of addressing the main points that can lead to the FBD, and all items included were considered important and comprehensive by the experts (both with agreement by Kendall coefficient ≥0.7—individually and per block). Additionally, they suggested specifying items 1.5, 1.6, and 1.7 better, where “Constructed of material that facilitates its cleaning” was replaced by “Constructed of material that facilitates its cleaning (plane, without roughness and cracks)”.

Item 2.4 (Equipment, furniture, and utensils hygiene) remained after the review (Delphi II) since kitchen utensils, cutting boards, dishcloths, and sponges become heavily contaminated with a diverse array of microbes, harboring and spreading contamination to hands, kitchen equipment, and contact surfaces. Although many handlers report cleaning these items after their use, studies indicated that most of the household handlers do not clean utensils sufficiently to prevent cross contamination [38,39,40]. Regarding the equipment’s conservation (item 2.1), the experts judged that it was enough to mention “in proper functioning” regardless of the temperature, since the equipment in Brazil do not have thermometers.

The item 4.2 (Food storage) included the expressions “Ingredients that have not been fully utilized are stored in clean, closed containers.” and “Ingredients that have not been fully utilized present the new date of shelf-life identification after opening, according to the manufacturer”. The “secure storage” keeps raw ingredients separate from ready-to-eat foods; allows one to know the shelf life of the product; and could avoid the potential contamination from the storage environment. These points are very important to reduce the chance of household FBD [37,38].

Despite the division of the instrument in blocks facilitating the instrument application, the physical structure, practices of food acquisition, storage, and manipulation should always be considered jointly so that the manipulative education investments based on perceptions or attitudes do not run counter to everyday domestic conditions [41].

To the best of our knowledge, there is no regulation for food preparation, handling, or storage in households. Many foodborne illness cases and their associated economic costs may be the result of preventable food handling mistakes at home [37]. Since most FBD cases are considered to be sporadic, mild, and unreported, it is estimated that the cases originating from food handling errors at home is much higher than we know [37]. Moreover, considering increasing concerns about the increase in cases of households’ foodborne diseases, it is urgent to encourage consumers to develop good food preparation practices at home. Household handlers are less likely to take protective steps when they place less importance on their own responsibility than that of others in the food safety chain. Helping consumers to understand the importance of the control they have in their homes as food safety risk managers can promote behavioral changes and reduce FBD. The development and validation of an instrument with objective criteria allows for the evaluation of the hygiene and safety conditions of the food in domestic environments, besides the perception of the handlers. Moreover, from an objective assessment, it is possible to evaluate the risks to the consumers, mainly the most vulnerable, such as immunosuppressed patients with various cancers, children, elderly and users of social programs [42].

Providing preventive information is an important strategy for reducing FBD cases because the aim of this information is to improve food handling practices in household environments [10]. The developed check-list presents strong points, since it was submitted to the evaluation of experts in the area and focus group who were free to make any comments relevant to improve the instrument. Moreover, the semantic evaluation process helped to ensure that the items were clear and comprehensive as to the language and writing [26]. The proposed check-list is attractive for its practicality and it can be used for identifying inappropriate household routines, allowing the correction of non-conformities, to ensure safe food.

## 5. Conclusions

Unsafe practices in food handling and consumption in the home environment have led researchers to assess the level of consumer knowledge about food and waterborne diseases and their consequences, as well as the interest in raising the awareness of food handlers about attitudes that may pose the risk of contamination of food in households. The use of an instrument as an evaluation tool facilitates the assessment of the level of food safety at home. However, in the scientific literature there is a shortage of easily reproducible validated instruments aimed at the planning of food safety education strategies, which could also be used to evaluate the results of training programs by food safety of health professionals. Therefore, we validated the instrument (check-list) developed for the verification of good practices in home kitchens, with respect to content and comprehension, after careful revision of its items. After it was redesigned, the items were considered important and comprehensive by the experts and focus group to Household Food-Borne Disease Assessment and Prevention. It is important to highlight that future studies are necessary to assess other properties of the instrument, such as reliability using the criteria of reproducibility, which aims at verifying the proportion of agreement among the responses when the instrument is applied in the same location and circumstances by different people. Further studies are also necessary to test this instrument in households and to evaluate its effectiveness in contributing to the prevention of food contamination. Strategies such as this are very important to improve the access to safe food.

## Figures and Tables

**Table 1:** Experts’ evaluation of the instrument, mean grades of the items, and the Kendall coefficient (*W*) of the section of the check-list. Brasília, DF, 2019.

Construction and Facilities				
Section of the Check-List	Content Validation (Mean Grade ± SD *)	Content Validation (*W*-Values)	Semantic Validation (Mean Grade ± SD *)	Semantic Validation (*W*-Values)
Outdoor area	3.86 ± 0.38	0.86	3.69 ± 0.48	0.70
Indoor area	3.86 ± 0.38	0.86	3.62 ± 0.50	0.70
Kitchen floor	3.71 ± 0.49	0.71	3.77 ± 0.44	0.77
Kitchen ceiling	4.00 ± 0.00	1.00	3.62 ± 0.50	0.70
Walls and kitchen divisions	4.00 ± 0.00	1.00	3.62 ± 0.50	0.70
Kitchen door	3.86 ± 0.38	0.86	3.54 ± 0.52	0.70
Windows and other kitchen openings	4.00 ± 0.00	1.00	3.69 ± 0.48	0.70
Toilets	3.86 ± 0.38	0.86	3.92 ± 0.28	0.92
Lighting and kitchen electrical wiring	3.86 ± 0.38	0.86	3.62 ± 0.50	0.70
Ventilation and acclimatization system of the kitchen	3.71 ± 0.49	0.71	3.69 ± 0.48	0.70
Urban vector and pest control	3.57 ± 0.53	0.70	3.77 ± 0.44	0.77
Water supply	3.57 ± 0.53	0.70	3.77 ± 0.44	0.77
Waste management	3.86 ± 0.38	0.86	3.77 ± 0.44	0.77
Sanitary sewage	3.57 ± 1.13	0.86	3.62 ± 0.50	0.70
Total of the block	3.88 ± 0.46	0.87	3.69 ± 0.48	0.70
Equipment, furniture, and kitchen utensils				
Equipment	4.00 ± 0.00	1.00	3.62 ± 0.50	0.70
Furniture	4.00 ± 0.00	1.00	3.69 ± 0.48	0.70
Utensils	3.86 ± 0.38	0.86	3.92 ± 0.28	0.92
Equipment, furniture, and utensils hygiene	4.00 ± 0.00	1.00	3.77 ± 0.44	0.77
Total of the block	3.92 ± 0.22	0.98	3.77 ± 0.44	0.77
Handlers				
Hygiene habits	3.86 ± 0.38	0.86	3.92 ± 0.28	0.92
Health condition	3.86 ± 0.38	0.86	3.85 ± 0.37	0.85
Total of the block	3.86 ± 0.38	0.86	3.96 ± 0.27	0.93
Food and feedstock				
Food and feed stock origin	4.00 ± 0.00	1.00	3.92 ± 0.28	0.92
Food storage	4.00 ± 0.00	1.00	3.92 ± 0.28	0.92
Total of the block	4.00 ± 0.00	1.00	3.92 ± 0.28	0.92
Total of the instrument	3.86 ± 0.38	0.86	3.62 ± 0.50	0.70

* Standard-deviation.

## Appendix A

**Good Practices in Home Kitchens: Checklist**

**Address: ______________________________**

**Age (years):__________ Job:_________________________________ Scholarship level: ______________ Number of people who live in household:_________________**

**Family income: _______________________**

**Presence of (in household): Elderly ( ) Pregnant ( ) Child ( )**

**Date: _____/______/________**

**Check-list to evaluate home kitchens**

Evaluation	**Y**	**N**	**NA**
1. Construction and Facilities			
1.1 External Area			
External area free of outbreaks of unhealthiness, garbage accumulation, stagnant water, among others.			
Access roads with adequate sewage system.			
1.2 Internal Area			
Kitchen free of obsolete objects or strangers to the environment.			
Free of the presence of domestic animals.			
1.3 Floor			
In good state of cleanliness.			
Cleaned at least once a day.			
Floor in material that facilitates its cleaning (ceramics or similar).			
In suitable state of conservation (free of defects, cracks, cracks, holes, and others).			
1.4 Kitchen Ceiling			
With appropriate ceiling material that facilitate cleaning (plaster, pvc, concrete, or similar).			
In suitable state of conservation (free of cracks, humidity, mold, fungus, spider webs, paint peel, and others).			
1.5 Walls and Kitchen Divisions			
In good state of cleanliness.			
Built with material of easy cleansing.			
In suitable state of conservation (free of cracks, humidity, paint peel, and others).			
1.6 Kitchen Door			
In suitable state of conservation (free of cracks, humidity, paint peel, and others).			
Built with material of easy cleansing.			
1.7 Windows and Other Kitchen Openings			
In suitable state of conservation (free of cracks, humidity, paint peel, and others).			
Built with material of easy cleansing.			
1.8 Toilets			
Toilet with intact toilet seat and with lid.			
Toilet with running water and connected to the sewage system or septic tank.			
Toilet without direct link to the kitchen area and/or the dining room.			
Toilet with bins with lids and pedal triggering.			
1.9 Lighting and kitchen electrical wiring			
Recessed lighting or when external, covered by insulating pipes attached to walls and ceiling.			
Bulbs and electric switches free of dirt.			
Cleaning of lamps, outlets, and electric switches at least once a month.			
1.10 Ventilation and Acclimatization System of the Kitchen			
Kitchen with ventilation and air circulation capable of thermal comfort.			
Kitchen free of fungus, causing no harm to food.			
1.11 Urban Vector and Pest Control			
Absence of urban vectors and pests or other evidences such as feces, nests, and other.			
Disinfecting every six months.			
1.12 Water Supply			
Water supply system connected to the public grid.			
Proper, protected, covered, and distant from contamination water system.			
Water tank with lid and in good state.			
Cleaning of water tank every six months.			
1.13 Waste Management			
Easy to clean and carry waste bins; in proper state and with appropriate garbage bags.			
Covered waste bins with pedal triggering.			
No waste bins over the sink.			
Waste stored in appropriate areas.			
1.14 Sewage System:			
Septic tank and sewage system connected to the public sewage.			
Observations:			
2. Equipment, Furniture, and Kitchen Utensils.			
2.1 Equipment:			
Evaluation	Y	N	NA
Fridge and stove in an area that permits adequate cleaning.			
Conservation equipment for food (fridges, freezers, and others) in proper functioning.			
Thermal food equipment (stove, oven, and/or microwave) in proper functioning.			
2.2 Furniture (tables, countermiddle, cupboard, shelves)			
From resistant material with proper surface conditions.			
Withdrawing that allows easy cleaning (smooth, without roughness, and chinks).			
2.3 Utensils:			
Size and shape for easy cleaning and in proper state.			
No wooden utensils or other materials of easy contamination.			
Pans, pots, and trays in proper state.			
Boards, knives, skimmers, and holders in proper state.			
Slicers and squeezers in proper state.			
Utensils (plates, silverware, bowls) in proper state.			
2.4 Equipment, Furniture, and Kitchen Utensils Cleaning			
Fridges and freezers in proper state.			
Fridges or freezers cleaned at least once a week.			
The stove is cleaned when used.			
Dishcloths in proper and cleaned state.			
Dishrags or table rags in proper state.			
Dishcloths are changed daily.			
Dishrags are changed daily.			
Cleaning sponges in proper hygiene and state.			
Cleaning sponges changed weekly.			
Cleaning products approved by the health department.			
Cleaning products in their original packing and stored in proper location.			
Equipment, furniture, and kitchen utensils in proper hygiene and state.			
Water filters changed every six months.			
No sponges from steel or wool.			
Observations:			
Evaluation	Y	N	NA
3. Handlers			
3.1 Hygiene Habits			
Personal cleaning, good appearance, clean hands, short nails, and unadorned (rings, earrings, bracelets, others).			
Handlers with previous knowledge on hand washing.			
3.2 Health Condition			
Absence of skin rash, wound, suppuration; absence of respiratory, eye, and gastric infections.			
Observations:			
Evaluation	Y	N	NA
4.0 Food and Feedstock			
4.1 Food and Feedstock Origin			
Food and feedstock with labels and packaging according to legislation.			
Milk from a secure source.			
Cheese from a secure source, packed and labeled.			
Meat, chicken, or fish from proper establishments.			
Filtered or boiled water consumption.			
4.2 Food Storage			
Semi perishable food stored in adequate and organized area with air circulation and lighting.			
Food prepared in advance before serving heated again.			
Fridges and freezers organized in order to avoid cross contamination.			
Feedstock not used completely are properly stored in clean and closed container.			
Feedstock not used completely are identified with expiration date.			
Perishable food stored in adequate temperature.			
Packages well cleaned before used for fridge or freeze storage.			
Leftovers stored under refrigeration and with bowls with lids.			
Observations:			
